# Stem Cells from the Apical Papilla (SCAPs): Past, Present, Prospects, and Challenges

**DOI:** 10.3390/biomedicines11072047

**Published:** 2023-07-20

**Authors:** Qi Liu, Yuan Gao, Jinzhi He

**Affiliations:** 1 State Key Laboratory of Oral Diseases & National Clinical Research Center for Oral Diseases, Sichuan University, Chengdu 610041, China; 2 Department of Cariology and Endodontics, West China Hospital of Stomatology, Sichuan University, Chengdu 610041, China

**Keywords:** mesenchymal stem cells, stem cells from apical papilla, regenerative endodontic treatment

## Abstract

Dental diseases occurring on young permanent teeth usually lead to the premature arrest of tooth root development. Sustained tooth root elongation is necessary to achieve the goal of long-term preservation of affected teeth. To this end, stem cell-based regenerative endodontic treatment has been regarded as one of the most promising strategies for treating young permanent teeth with pulp and periapical infections. Endogenous stem cells residing in the apical papilla, named stem cells from the apical papilla (SCAPs), have been intensively investigated due to their critical roles in pulp regeneration and root redevelopment. The present review summarizes advances in the field of SCAPs studies and discusses the challenges that need to be further addressed.

## 1. Introduction

The tooth root is the invisible two-thirds of a tooth embedded in the jaw bone and responsible for anchoring the tooth. Without the tooth root, the crown cannot fulfill its physiological functions. Tooth root development initiates immediately after crown formation [1]. When enamel reaches the cementum–dentin junction, the apical region of the enamel organ elongates and forms a bilayer epithelial structure named the Hertwig’s epithelial root sheath (HERS). The HERS is closely surrounded by cranial neural crest-derived mesenchymal tissues, which can be further divided into the dental papilla and dental follicle [2]. During tooth root formation, the HERS works as a signal center, from which the dental papilla and dental follicle receive signals to guide the generation of root tissues [1]. During tooth root development, mesenchymal stem cells (MSCs) from the dental papilla and dental follicle undergo strict, lineage-specific differentiation programs. Specifically, MSCs from the dental papilla differentiate into odontoblasts (which are responsible for root dentin formation) and dental pulp cells [3]. MSCs residing in the dental follicle contribute to the formation of cementoblasts, osteoblasts, and fibroblasts, which produce cementum, alveolar bone, and periodontal ligament surrounding the newly developed tooth root, respectively [3].

The tooth erupts before root development finishes. In humans, it usually takes 3–5 years for the roots of newly erupted teeth to reach their full length and thickness. Before tooth root development completes, the tooth is called a young permanent tooth. Not all young permanent teeth are able to develop roots with normal anatomic structures. Diseases with a high prevalence, including dental caries, dental trauma, and tooth dysplasia, usually lead to pulpal and periapical infection in young permanent teeth. This infection causes not only the loss of pulpal vitality and the destruction of periapical tissues, but also the premature arrest of tooth root development. In this case, tooth root elongation and radicular dentinogenesis cease, resulting in a shortened tooth root with thin dentinal walls and a wide-open apex because of the dysfunction of mesenchymal stem cells (MSCs) in both the dental papilla and dental follicle. Traditional treatment techniques, such as apexification and apical barrier surgery, although effective in controlling infection, cannot restart the development of the tooth root [4]. In other words, disharmony in the crown root ratio cannot be rescued and the thickness of the root canal wall cannot be increased using these treatments, which jeopardizes the long-term preservation of affected teeth [5,6]. The successful treatment of young permanent teeth with pulp and periapical infection must include sustained root development.

In recent years, stem cell-based regenerative endodontic treatment (RET) has been adopted as a promising strategy for treating young permanent teeth with pulp and periapical infection [7]. The goal of RET is to achieve the continued development of the tooth root as well as the functional regeneration of the pulp–dentin complex [8]. RET can generally be divided into cell-based RET, based on the principle of exogenous stem cell transplantation, and cell-free RET, based on the principle of endogenous stem cell homing [9]. Compared to cell-based RET, cell-free RET realizes the goal of tooth root redevelopment through inducing the accumulation of endogenous MSCs at the lesion site, avoiding both technical and ethical problems that stem cell transplantation needs to solve [9]. Based on the studies released so far, stem cells from the apical papilla (SCAPs) are among the most promising endogenous MSCs to achieve the goal of RET in young permanent teeth losing pulpal vitality [8]. In the present review, advancements in the field of SCAPs are summarized, and the challenges of further investigation are discussed. This review might shed light on the current understanding of the clinical translational potential of SCAPs and elucidate possible areas for future research applications.

## 2. Discovery of SCAPs, the Stem Cells Residing in the Apical Papilla

The apical papilla is, essentially, the apical part of the dental papilla. Histologically, the apical papilla can be seen precisely apical to the HERS, with a cell-rich zone separating it from the dental pulp [10]. In 2006, Sonoyama et al. collected apical papilla tissues from the root end of a young human third molar and then cultured cells from this tissue. They noticed, for the first time, that isolated cells exhibit classical MSCs properties, including clonogenic cell cluster formation and odontoblastic/osteoblastic differentiation [11]. The identified cells were then named stem cells from the apical papilla (SCAPs). Evidence strongly supporting the existence of SCAPs is that root maturation ceases to continue in the absence of the apical papilla [10]. Other robust evidence is from in vivo transgenic animal studies. Although mouse incisors have no root, their molars are quite like human molars. The similarity makes the mouse molar a good model for us to understand the biology of human tooth root development. With transgenic mouse lines and the well-established lineage tracing method, Feng et al. found that Gli1-expressing cells in the most apical part of the dental papilla and dental follicle contribute to the formation of the dentin, pulp, and periodontal tissue of the root [12]. In addition, Ono detected another group of SCAPs, namely, osterix-expressing cells in the apical part of developing mouse molars, including dental mesenchymal progenitors that contribute to the formation of all cell types involved in further dental root development [13]. Furthermore, Jing et al. collected mouse molars at different root developmental stages and analyzed them using single cell RNA sequencing technology [14]. They confirmed that postnatal apical papilla retains bipotent progenitor cells that give rise to both dental pulp cells and odontoblasts during mouse molar development [14]. These pioneer studies not only support the existence of SCAPs, but also show how transgenic mouse lines are useful for investigating the biology and the regulation of SCAPs in vivo.

## 3. Culture-Dependent Methods and SCAPs Studies

The most widely used strategy for the study of SCAPs, thus far, is culture dependent. The prevalent apical papilla tissues used for culturing are collected from human teeth, especially wisdom teeth. The apical papilla can be easily isolated following tooth extraction by separating the tissues at the tips of developing roots with tweezers. Then, the tissue is dissected into smaller pieces and digested using a cocktail of collagenase and dispase to prepare single-cell suspensions for culturing. Meanwhile, the dissected tissues can be also used directly for culturing without digestion, and this method is known as explant culture. Isolated SCAPs can be preserved in nitrogen until use, and cryopreservation does not affect the biological and immunological properties of SCAPs [15].

During the literature review, we noticed that, at present, the majority of findings on SCAPs are from in vitro studies. Although the culture-dependent method provides us with useful clues for understanding the biology of this cell population, when interpreting the findings, some key points are necessary to keep in mind. First, cultured SCAPs, especially at late passage, demonstrate a transcriptome and phenotypes that are distinct from those of their counterparts in native tissues [16,17]. Second, cellular phenotypes can be affected by the genetic background of the donors, the tooth development stage, the culture condition, the generation of cell lines, etc. For example, SCAPs from teeth in the early root development stage showed better cell migration and osteogenic differentiation than those isolated from the tooth root at the late stage of development [18]. Third, the role of SCAPs in tooth root development is regulated by their in vivo niche, and in vitro culturing inevitably simplifies the regulation network SCAPs receive [19]. Therefore, different, even opposite, findings might be observed in studies from different research groups.

## 4. Characteristics of SCAPs as Typical Mesenchymal Stem Cells

Morphologically, SCAPs are small and fibroblast-like or stellate in shape [20]. SCAPs express a range of typical MSC-associated markers, including STRO-1, CD146, CD24, CD29, CD73, CD105, CD106, CD166, and CD90, but are negative for the surface molecules CD18, CD14, CD34, CD45, and CD150 (all of which indicate a hematopoietic origin) [11,21,22]. However, it should be kept in mind that all the markers mentioned above are nonspecific markers for SCAPs. For example, STRO-1, CD146, CD73, and CD105 can also be detected in another group of dental mesenchymal stem cells, that is, dental pulp stem cells (DPSCs) [20,23,24]. Therefore, to distinguish SCAPs and DPSCs, different phenotypes need be taken into consideration. *In vitro*, SCAPs have cytoplasmic extensions named filopodia, exhibiting a spindle-shaped which is found in narrow to large polygonal cells, while DPSCs are larger in size [20]. In addition, SCAPs show a significantly higher proliferation rate, mineralization rate, and migration ability than do DPSCs [20]. It has been shown that heterogeneity exists in mesenchymal stem/progenitor cell pools within the dental papilla [25]. Similarly, SCAPs might also be a heterogeneous population containing distinct subsets of stem/progenitor cells with different cellular characteristics. Studies also prove that SCAPs are capable of proliferation and multilineage differentiation, including odontogenic, osteogenic, neurogenic, chondrogenic, and adipogenic differentiation [11,26].

## 5. Maintenance of Cell Vitality by SCAPs in Dental Inflammation

Despite the dental pulp and apical papilla being a continuity of one another, when necrosis spreads in the pulp and the periapical tissues, the apical papilla tends to survive. By evaluating the histopathological conditions of the dental pulp and apical papilla after inducing endodontic infection in a rat model, Tobias Duarte et al. noticed that the apical papilla of an immature tooth remained vital, yet slightly or moderately inflamed, after 90 days of infection, although pulp necrosis and the following apical periodontitis occurred as early as 14 days of infection [27]. Another animal study introduced endodontic infection to the immature canine molars of dogs, and the apical papilla was found to be present and histologically distinct from other tissues despite the advanced pulpal infection and endodontic procedures [28]. These findings strongly suggest that the apical papilla provides a relatively stable environment for SCAPs to keep their vitality and stemness. This hypothesis is strongly supported by clinical observations. The tooth root development of young permanent teeth terminated when the pulp and periapical were infected. However, the affected tooth root resigned to elongation/development after the inflammation was well controlled. In addition, a study harvested inflamed periapical tissue from a human immature mandibular premolar diagnosed with pulp necrosis and apical periodontitis [29]. The survival of the apical papilla and its resident stem cells was observed, elucidating the infection-resistant nature of SCAPs. Potential reasons for the survival of SCAPs through endodontic inflammation include, first, dental MSCs being usually distributed in areas surrounding the neurovascular bundle [30,31], and their proximity to the periapical vasculature that gives SCAPs the advantage of survival in a detrimental environment. Second, histologically, the apical papilla has a quite lower number and density of blood vessels compared to the neighboring dental pulp [32,33], suggesting that cells within the dental papilla, including SCAPs, have low metabolic demands when in the quiescent stage. Third, the apical papilla has intimate contact with the dental follicle, the most vascularized dental tissue. Therefore, dental papilla cells may acquire nutrient and gaseous exchanges diffused from the dental follicle when inflammation involves the apical papilla [33].

## 6. In Vivo Niche of SCAPs

The surrounding environment in which a stem cell resides is called its niche [34]. The niche is not only composed of stem cells and their progenies, but multiple heterologous cell types as well as a niche-specific extracellular matrix [35]. The niche is a key regulator of stem cells, as it provides signals to stem cells to mediate their rate of proliferation, determines the fate of their daughters, and protects them from exhaustion or death [35]. The signals can be soluble molecules secreted from the stem cells themselves, from neighboring niche cells, or from other tissues. And the signals can be also from extracellular matrices or mechanical force.

The apical papilla is the niche of SCAPs. To the best of our knowledge, there is no study available that specifically shows its cellular compositions. Importantly still, the function of these niche cells, as well as how they incorporate with each other to orchestrate the behavior of SCAPs, is still not clear. In recent years, single-cell RNA sequencing and spatial transcriptome profiling techniques have been widely used to explore cell types as well as their in vivo distributions within different tissues [36,37]. Hopefully, this technology can help us identify clues to answer the aforementioned questions. In addition to the cells, other factors of the SCAPs niche are under explored. For example, cytokines are crucial chemical signals in stem cell niches. They are small proteins produced and secreted by cells. Cytokines can bind to cell surface receptors, triggering gene cell expression and regulating the growth, maturation, and responsiveness of cell populations. Although a comprehensive cytokine profile of the apical papilla is absent, in vitro studies have shown that SCAPs are able to synthesize various cytokines, such as IGF-1, IGFBP-6, IL-10 [38], and TGF-β3 [39]. The roles these cytokines play in regulating the cellular behaviors of SCAPs in vivo are still unknown although some in vitro studies, as discussed later in this review, have already be carried out.

## 7. Regulation of SCAPs by Signaling Molecules and Pathways

The process of tooth root development and elongation is finely tuned by signaling molecules and their downstream pathways, such as the transforming growth factor-β superfamily, the fibroblast growth factor, Wnts, and Shh [1]. Studies have shown that these signaling molecules and their downstream pathways influence the length of the tooth root through regulating the MSCs or progenitors responsible for tooth elongation [1]. At the same time, the effects of these signaling molecules, as well as their down-stream pathways, on SCAPs have been intensively tested, mainly *in vitro*. Briefly, the majority of these studies have focused on their influence on the proliferation, migration, and differentiation of SCAPs, as summarized in Table 1.

### 7.1. Transforming Growth Factor-Β Superfamily

The TGF-β superfamily consists of TGF-β 1-3, bone morphogenetic proteins (BMPs), activins, inhibins, Müllerian-inhibiting substances (MISs), nodals, growth and differentiation factors (GDFs), and the distantly related glial cell line-derived neurotrophic factor (GDNF) family [40]. The binding of TGF-β to its receptors, including type II (TβRII) and type I (TβRI) receptors, leads to formation of the receptor heterocomplex and the activation of TβRI. The activated TβRI recruits and phosphorylates R-Smad proteins (Smad2/3 for TGF-β and activin signaling while Smad1/5/8 for BMP signaling), and then the phosphorylated R-Smad forms a heterocomplex with the Co-Smad Smad4. The Smad complexes are then translocated into the nucleus, where they cooperate with other cofactors and regulate the transcription of the target genes. SCAPs express the TGF-β receptor 1 [10], suggesting they can receive regulation from TGF-β ligands. Secreted TGF-β1 and TGF-β2 are embedded in the dentin during tooth development, and they can release from the dentin during endodontic procedures, such as root canal therapy [41,42,43]. Studies have demonstrated that TGF-β1 can inhibit the proliferation, differentiation, and mineralization of SCAPs in dose-dependent and Smad3-dependent manners [41,44]. Interestingly, TGF-β1 inhibits the odontogenic differentiation of SCAPs while enhancing their osteogenic differentiation. Conversely, TGF-β2 attenuates the osteogenic differentiation of SCAPs but promotes the odontogenic differentiation of SCAPs [45,46]. These results suggest that the ratio between TGF-β1 and TGF-β2 is important in regulating the fate of SCAPs. Other proteins in the TGF-β superfamily also participate in the regulation of SCAPs. For example, BMP2 promotes SCAPs differentiation into odontoblasts [47,48], while BMP6 enhances not only the osteo/dentinogenic differentiation of SCAPs but also their neurogenic and angiogenic differentiation and proliferation [49].

### 7.2. Wnts

Wnts are highly conserved, lipid-modified, secreted proteins. To date, 19 different mammalian Wnt proteins have been identified in humans and mice. Wnt proteins activate signaling by interacting with their receptors, called Frizzled. A combinatorial interaction between different Wnt proteins and Fz receptors results in the activation of multiple downstream pathways, which are classified into two broad categories: the β-catenin dependent canonical Wnt pathway and the β-catenin independent non-canonical Wnt pathways. The activity of Wnt proteins can be inhibited by several antagonists that bind either to the Wnt ligand itself (e.g., Wnt Inhibitory Factor-1 (WIF-1), and secreted Frizzled-Related Proteins (sFRPs)), or to Wnt receptor (e.g., Dickkopf (Dkk) proteins)). Treatment with exogenous Wnt-1 increases both the proliferation and differentiation of SCAPs through activating the canonical Wnt pathway [50]. Unexpectedly, Wnt antagonists also have similar influence on the differentiation of SCAPs. Specifically, WIF-1 enhances the dentinogenic differentiation of SCAPs [17], and Sfrp2 increases not only the osteo/odontogenic abilities of SCAPs but also their adipogenic and neuronal differentiation abilities [26,51,52], especially under inflammation and hypoxia conditions [52]. These inconsistent findings regarding the effects of Wnt and its mediated signaling pathway on SCAPs require further investigation. In addition, Wnt5a, a typical ligand of the noncanonical Wnt pathway, suppresses the osteogenesis of SCAPs [53]. These findings suggest the distinct role of canonical and non-canonical Wnt pathways in regulating SCAPs.

### 7.3. Fibroblast Growth Factors (FGFs)

The FGF family comprises twenty-two ligands that interact with four FGF receptors (FGFRs). Signaling from FGFs is a key regulator of stem cell pluripotency [54]. FGFR1 and FGFR2 express on SCAPs, suggesting that SCAPs can be regulated by FGFs molecules [10,55]. Basic fibroblast growth factor (bFGF), a type of FGF, increases proliferation and colony-forming unit formation [55,56], as well as the expression of stem cell gene markers, such as STRO-1, in SCAPs [56]. In addition, bFGF also effectively stimulates the migration of SCAPs [57]. Further studies have shown that bFGF mediates the cell behaviors of SCAPs through the stimulation of FGFRs and the MEK/ERK signaling pathway [55,58].

### 7.4. Insulin-like Growth Factors (IGFs)

IFGs mediate signaling pathways that participate in tooth root determination [59]. There are two IGFs, including IGF-1 and IGF-2. They share a 70% homology in the amino acid sequence. SCAPs express the type 1 IGF-1 receptor (IGF-1R), suggesting they might be regulated by IGFs. Co-culturing with IGF-1 leads to morphology changes in SCAPs. In addition, IGF-1 enhances the proliferation [59], osteogenic differentiation [59], and both glial and neuronal differentiation [60] of SCAPs but weakens their odontogenic differentiation [59]. Similarly, IGF-2 promotes the osteo-/dentinogenic and neurogenic differentiation potentials of SCAPs [61]. A further mechanistic analysis demonstrated that IGF-1 activated AKT to regulate the early neural differentiation of human SCAPs [60].

### 7.5. Other Signaling Molecules

Vascular endothelial growth factor (VEGF) is important in revascularization procedures because it stimulates the process of angiogenesis. The overexpression of VEGF increases the proliferation of SCAPs [62]. In addition, VEGF shows no effect on viability- and dentinogenesis-related gene expression in SCAPs [63]. If SCAPs are first cultured with LPS, VEGF can stimulate the expression of dentinogenesis-related genes in SCAPs, including *DSPP*, *DMP1,* and *TGFB1* [63].

Nerve growth factor is a neurotrophic molecule that plays a vital role in the growth and development of neurons [64]. NGF treatment promotes the expression of dentinogenesis-related genes in SCAPs, including *DSPP*, *DMP1,* and *TGFB1* [63]. Interestingly, in SCAPs that have been previously treated with LPS, NGF shows an opposite effect [63].

Angiotensin II (Ang II) is the primary effector peptide of the renin–angiotensin system (RAS) [65], and it has been detected in oral tissues, including the periodontal ligament, gingiva, and dental pulp. The Ang II peptide is also detectable from the primary cultures of SCAPs supernatants [66]. Exogenous Angiotensin II supplementation significantly enhances SCAPs proliferation and CCL2 production while inhibiting IL-6 release and the mineralization rate of SCAPs [66].

### 7.6. Synergism and Antagonism among Signaling Molecules in Regulating SCAPs

Synergistic and antagonistic effects between signaling molecules have also been tested. As an example, BMP2 does not affect the proliferation of SCAPs while VEGF treatment promotes proliferation and BMP2 expression in these cells [62]. When increasing the expression levels of these two hormones simultaneously in SCAPs, proliferation is inhibited while the osteo-/odontogenic differentiation of human SCAPs is enhanced [62]. In addition, synergistic effects of stromal cell-derived factor-1α and BMP2 on odontogenic differentiation also have been detected in SCAPs cultured using the Vitro-Gel 3D system [67].

**Table 1:** Effect of signaling molecules on the cellular behavior of SCAPs.

	Molecules	Roles in Regulating SCAPs	Refs.
TGF-β superfamily	TGF-β1	proliferation (-), odontogenic differentiation (-), osteogenic differentiation (+), mineralization (-)	[41,44,45,46]
	TGF-β2	osteogenic differentiation (-), odontogenic differentiation (+)	[45,46]
	BMP2	odontogenic differentiation (+)	[47,48]
	BMP6	osteo/dentinogenic differentiation (+), neurogenic differentiation (+), angiogenic differentiation (+), proliferation (+)	[49]
Wnt	Wnt-1	proliferation (+), differentiation (+)	[50]
	WIF-1	dentinogenic differentiation (+)	[17]
	Sfrp2	osteo/odontogenic differentiation (+), adipogenic differentiation (+), neuronal differentiation (+)	[26,51,52]
	Wnt5a	osteogenesis (-)	[53]
FGF	bFGF	proliferation (+), colony-forming unit formation (+), migration (+)	[55,56,57]
IFG	IGF-1	proliferation (+), osteogenic differentiation (+), glial and neuronal differentiation (+), odontogenic differentiation (-)	[59,60]
	IGF-2	osteo-/dentinogenic differentiation (+), neurogenic differentiation (+)	[61]
Others	VEGF	proliferation (+)	[63]
	NGF	dentinogenesis (+)	[63]
	Ang II	proliferation (-), mineralization (+)	[66]

(-) indicates inhibiting effect, (+) indicates promoting effects.

## 8. Interactions between SCAPs and Oral Bacteria

Infection by oral microbiota is a predominant reason for inflammation on the apical papilla and the termination of tooth root elongation. When infection occurs, the microbes and their components get the chance to go into the apical papilla. Consequently, SCAPs become close to oral microbiota and microbial components, affecting their physiology directly. Moreover, the invaded pathogens create an inflammatory environment with immune cell infiltration and inflammatory cytokine accumulation. The altered stem cell niche inevitably causes changes in the cellular behaviors of SCAPs.

Bacteria and their cellular components (such as lipoproteins (LPS), lipoteichoic acid (LTA) and peptidoglycans) can directly bind to and then activate receptors on mammalian cells, such as Toll-like receptors (TLRs). So far, 10 functional TLRs have been characterized in human cells, and the expression of TLRs is ubiquitous and can be detected on MSCs. Accordingly, SCAPs demonstrate a unique expression profile out of all TLRs 1–10 [68], and the expression patterns of TLRs can be influenced by secreted products of both oral planktonic (free-moving) bacteria and oral biofilms (a well-organized microbial community attached to the tooth structure). For example, incubation with the soluble extracellular products of the endodontopathogenic bacteria *Streptococcus oralis* and *Actinomyces naeslundii,* specifically, upregulates TLR2 expression on SCAPs [69]. The upgraded TLR2-mediated TLR2-TAK1 is a key pathway through which bacteria block the mineralization capacity of SCAPs [69].

Microbes regulate the viability, proliferation, mineralization/differentiation, cytokine secretion, and key signaling pathways of SCAPs via their cellular components and products directly [69,70,71,72,73,74]. The soluble extracellular products of *Streptococcus oralis* and *Actinomyces naeslundii* have a detrimental effect on the viability, proliferation, and mineralization/differentiation capacity of SCAPs [69]. LPS produced from the gram-negative bacteria *Porphyromonas gingivalis* decreases the cell viability and differentiation potential of SCAPs, and the suppression of SCAP osteo-/odontogenic differentiation can be rescued via autophagy inhibition [70]. In addition, LPS induces the expression of the pro-inflammatory cytokines IL-1β and TNF-α in a dose-dependent manner [74]. It also activates canonical Wnt/β-catenin and p38 MAPK signaling in SCAPs [74]. A recently published study further analyzed how pre-exposure to *P. gingivalis* LPS affects the proliferation, migration, and osteogenic differentiation of SCAPs isolated from the root at the early and late development stages. They found that pre-exposure to *P. gingivalis* LPS did not affect the proliferation and migration of SCAPs. Interestingly, for SCAPs isolated from the apical papilla of a tooth root at the early development stage, pre-treatment with LPS increased its differentiation ability [18].

Endodontic pathogens also affect SCAPs in an indirect way. The invasion of bacteria into the root canal system and the apical papilla results in increased levels of immune cells and inflammatory cytokines. The inflammatory cytokines up-regulate the expression of TLR1, TLR2, TLR4, TLR5, TLR6, and TLR9 while significantly down-regulating the expression of TLR3, TLR7, TLR8, and TLR10 [68]. The altered TLR profile can then exert an effect on SCAPs. In addition, endodontic pathogens and their cell surface components, including LPS and LTA, can stimulate the production of TNF-α via macrophages, and TNF-α exerts deleterious effects on SCAPs by affecting their viability, proliferation rate, and mineralization potential [75].

The influence of oral microbiota on the biology of SCAPs is TLR, species, and culture dependent [69]. For example, *Fusobacterium nucleatum* triggers pro-inflammatory chemokine and cytokine generation in SCAPs while *Enterococcus faecalis* inhibits the same [73]. In addition, *E. faecalis*, but not *F. nucleatum,* can downregulate the expression levels of key proteins in Wnt/β-Catenin and NF-κB signaling pathways [76].

## 9. Effects of Dental Procedures on SCAP Biology

RET starts with the sterilization of the root canal system and the creation of a microenvironment suitable for the adhesion, survival, and differentiation of MSCs. There are mainly three methods for removing the microbiota invading root canal systems, including minimal mechanical preparation, chemical irrigation, and placement of intracanal medicament.

Mechanical preparation during root canal disinfection entails the removal of infected pulp and dentine from the root canal system via filing. During this process, a smear layer composed of dentine, remnants of pulp tissue and odontoblastic processes, and sometimes bacteria, is always formed on the canal walls. It is possible that the smear layer affects the cellular behavior of SCAPs due to its composition. However, this hypothesis has not yet been tested to the best of our knowledge.

The most widely used irrigants during endodontic therapy, including RET, are 17% ethylenediaminetetraacetic acid (EDTA), 0.5~6% sodium hypochlorite (NaOCl), 2% chlorhexidine (CHX), and saline. EDTA is a chelator, and its solution is neutral or slightly alkaline. This molecule has little or even no antibacterial effect. However, it chemically softens the root canal dentine, dissolves the smear layer, and opens the dentinal tubules. Additionally, 17% EDTA sustains SCAPs survival even better than saline [77]. Potential mechanisms for the protective effect of EDTA on SCAPs includes its chelating effect accelerating the release of dentin-derived growth factors (for instance, TGFs, as discussed above) that were previously embedded into dentin, and these growth factors fostering the survival of dental stem cells. NaOCl is an antiseptic lubricant that has been used in dilutions ranging from 0.5% to 5.25% during endodontic procedures. NaOCl, at concentrations ranging from 0.5~6%, showed detrimental effects on SCAPs survival that cannot be reversed by its neutralizer, sodium thiosulfate [77]. CHX is bacteriostatic at a concentration of 0.2% and bactericidal at a concentration of 2%. CHX at concentrations higher than 1% is cytotoxic to SCAPs, and 2% CHX treatment results in no viable SCAPs [78,79]. However, the deleterious effects of CHX can be completely reversed through neutralization with L-α-lecithin. During endodontic treatments, the combined use of irrigants is quite common. Different irrigation protocols have resulted in distinct SCAP survival rates. For example, the addition of a final irrigation with 17% EDTA reversed the negative effects of NaOCl on SCAPs survival [77]. However, 17% EDTA cannot reverse the deleterious effects of CHX [79].

The prevalent intracanal medicaments used in dental procedures include triple antibiotic paste (TAP, the mixture of ciprofloxacin, metronidazole, and minocycline), double antibiotic paste (DAP, the combination of metronidazole and ciprofloxacin), modified triple antibiotic paste (mTAP, the combination of metronidazole, ciprofloxacin, and cefaclor), and calcium hydroxide (Ca [OH]_2_). High concentrations of antibiotics have a detrimental effect on SCAPs survival, whereas antibiotic treatment at lower concentrations and Ca (OH)_2_ at all tested concentrations are conducive to SCAPs survival, proliferation, and differentiation [80,81,82].

During RET, a dental restorative material is applied directly to the blood clot. Bioceramic materials, in particular calcium silicate–based materials, have emerged as the top choice because of their biocompatibility, sealing, and antimicrobial potential. Bioceramic materials, such as mineral trioxide aggregate and Biodentine, have been proven to promote the survival, proliferation, and differentiation of SCAPs [64,83,84].

## 10. Challenges and Future Directions

In recent years, pulp revascularization, a treatment belonging to cell-free RET, has been widely used in clinics. In this approach, the root canal system is first disinfected with antibiotics or Ca [OH]_2_; then, it is filled with a blood clot from bleeding provoked in periapical tissues. The blood clot acts as a scaffold, and the growth factors inside the scaffold support the homing of MSCs and promote the continued development of the tooth roots. Case reports have shown that pulp revascularization increases both the root length and dentinal wall thickness of affected immature teeth. Although pulp revascularization can achieve high success rates in the treatment of young permanent teeth, this treatment still has shortcomings. Firstly, the therapeutic outcome of this technology is unstable, and the degree of root re-development varies across different studies. Secondly, histological analyses have shown that the tissues formed in root canals are translocated cementum, bone-like, and periodontal ligament-like connective tissues [85,86], suggesting a regeneration of the pulp–dentin complex has not yet been achieved. As early as 2011, Lovelace et al. discovered, for the first time, that in the process of regenerative pulp treatment, bleeding can lead to MSCs entering the root canal. These cells were found to be STRO-1 and CD105 positive, suggesting that they are SCAPs [87]. Subsequent studies also support the notion that SCAPs are the most important MSCs for achieving regenerative pulp treatment and root redevelopment. To overcome the shortcomings of cell-free RET, such as pulp revascularization, a comprehensive understanding of the biological characteristics and regulatory network of endogenous SCAPs in vivo is necessary. For quite a long time, due to a lack of reliable in vivo markers and transgenic mouse models, we know nothing about the in vivo spatiotemporal distribution, biological characteristics, or changes in cellular behavior and functions of SCAPs under inflammatory conditions, or about the related regulatory mechanisms. Therefore, it is quite urgent to screen in vivo makers and then establish repeatable transgenic mouse models in SCAPs studies.

## 11. Conclusions

SCAPs are regarded as the most promising endogenous MSCs to achieve the goal of RET in young permanent teeth with endodontic diseases. Gathering data has provided us with a chance to understand the properties of SCAPs. To maximize the clinical potential of SCAPs in RET, in vivo studies are necessarily required.

## Data Availability

Not applicable.

## References

[1] Li J., Parada C., Chai Y. (2017). Cellular and molecular mechanisms of tooth root development. Development.

[2] Orban B.J. (1980). Orban’s Oral Histology and Embryology.

[3] Krivanek J., Adameyko I., Fried K. (2017). Heterogeneity and Developmental Connections between Cell Types Inhabiting Teeth. Front. Physiol..

[4] Shabahang S. (2013). Treatment options: Apexogenesis and apexification. Pediatr. Dent..

[5] Simon S., Rilliard F., Berdal A., Machtou P. (2007). The use of mineral trioxide aggregate in one-visit apexification treatment: A prospective study. Int. Endod. J..

[6] Cvek M. (1992). Prognosis of luxated non-vital maxillary incisors treated with calcium hydroxide and filled with gutta-percha. A retrospective clinical study. Endod. Dent. Traumatol..

[7] Wikström A., Brundin M., Lopes M.F., El Sayed M., Tsilingaridis G. (2021). What is the best long-term treatment modality for immature permanent teeth with pulp necrosis and apical periodontitis?. Eur. Arch. Paediatr. Dent..

[8] Wei X., Yang M., Yue L., Huang D., Zhou X., Wang X., Zhang Q., Qiu L., Huang Z., Wang H. (2022). Expert consensus on regenerative endodontic procedures. Int. J. Oral Sci..

[9] Lin L.M., Huang G.T., Sigurdsson A., Kahler B. (2021). Clinical cell-based versus cell-free regenerative endodontics: Clarification of concept and term. Int. Endod. J..

[10] Sonoyama W., Liu Y., Yamaza T., Tuan R.S., Wang S., Shi S., Huang G.T. (2008). Characterization of the apical papilla and its residing stem cells from human immature permanent teeth: A pilot study. J. Endod..

[11] Sonoyama W., Liu Y., Fang D., Yamaza T., Seo B.M., Zhang C., Liu H., Gronthos S., Wang C.Y., Wang S. (2006). Mesenchymal stem cell-mediated functional tooth regeneration in swine. PLoS ONE.

[12] Feng J., Jing J., Li J., Zhao H., Punj V., Zhang T., Xu J., Chai Y. (2017). BMP signaling orchestrates a transcriptional network to control the fate of mesenchymal stem cells in mice. Development.

[13] Ono W., Sakagami N., Nishimori S., Ono N., Kronenberg H.M. (2016). Parathyroid hormone receptor signalling in osterix-expressing mesenchymal progenitors is essential for tooth root formation. Nat. Commun..

[14] Jing J., Feng J., Yuan Y., Guo T., Lei J., Pei F., Ho T.-V., Chai Y. (2022). Spatiotemporal single-cell regulatory atlas reveals neural crest lineage diversification and cellular function during tooth morphogenesis. Nat. Commun..

[15] Ding G., Wang W., Liu Y., An Y., Zhang C., Shi S., Wang S. (2010). Effect of cryopreservation on biological and immunological properties of stem cells from apical papilla. J. Cell. Physiol..

[16] Rémy M., Ferraro F., Le Salver P., Rey S., Genot E., Djavaheri-Mergny M., Thébaud N., Boiziau C., Boeuf H. (2019). Isolation and Culture of Human Stem Cells from Apical Papilla under Low Oxygen Concentration Highlight Original Properties. Cells.

[17] Wang H., Cao Y. (2019). WIF1 enhanced dentinogenic differentiation in stem cells from apical papilla. BMC Oral Health.

[18] Aguilar P., Mahanonda R., Sa-Ard-Iam N., Lertchirakarn V. (2021). Effects of lipopolysaccharide on proliferation, migration and osteogenic differentiation of apical papilla cells from early and late stage of root development. Aust. Endod. J..

[19] Driesen R.B., Gervois P., Vangansewinkel T., Lambrichts I. (2021). Unraveling the Role of the Apical Papilla during Dental Root Maturation. Front. Cell Dev. Biol..

[20] Bakopoulou A., Leyhausen G., Volk J., Tsiftsoglou A., Garefis P., Koidis P., Geurtsen W. (2011). Comparative analysis of in vitro osteo/odontogenic differentiation potential of human dental pulp stem cells (DPSCs) and stem cells from the apical papilla (SCAP). Arch. Oral Biol..

[21] Nada O.A., El Backly R.M. (2018). Stem Cells from the Apical Papilla (SCAP) as a Tool for Endogenous Tissue Regeneration. Front. Bioeng. Biotechnol..

[22] Kang J., Fan W., Deng Q., He H., Huang F. (2019). Stem Cells from the Apical Papilla: A Promising Source for Stem Cell-Based Therapy. Biomed. Res. Int..

[23] Aydin S., Şahin F. (2019). Stem Cells Derived from Dental Tissues. Adv. Exp. Med. Biol..

[24] Mattei V., Santacroce C., Tasciotti V., Martellucci S., Santilli F., Manganelli V., Piccoli L., Misasi R., Sorice M., Garofalo T. (2015). Role of lipid rafts in neuronal differentiation of dental pulp-derived stem cells. Exp. Cell Res..

[25] He J., Jing J., Feng J., Han X., Yuan Y., Guo T., Pei F., Ma Y., Cho C., Ho T.-V. (2021). Lhx6 regulates canonical Wnt signaling to control the fate of mesenchymal progenitor cells during mouse molar root patterning. PLoS Genet..

[26] Lin X., Dong R., Diao S., Yu G., Wang L., Li J., Fan Z. (2017). SFRP2 enhanced the adipogenic and neuronal differentiation potentials of stem cells from apical papilla. Cell Biol. Int..

[27] Tobias Duarte P.C., Gomes-Filho J.E., Ervolino E., Marçal Mazza Sundefeld M.L., TadahiroWayama M., Lodi C.S., Dezan-Júnior E., Angelo Cintra L.T. (2014). Histopathological Condition of the Remaining Tissues after Endodontic Infection of Rat Immature Teeth. J. Endod..

[28] Palma P.J., Ramos J.C., Martins J.B., Diogenes A., Figueiredo M.H., Ferreira P., Viegas C., Santos J.M. (2017). Histologic Evaluation of Regenerative Endodontic Procedures with the Use of Chitosan Scaffolds in Immature Dog Teeth with Apical Periodontitis. J. Endod..

[29] Chrepa V., Pitcher B., Henry M.A., Diogenes A. (2017). Survival of the Apical Papilla and Its Resident Stem Cells in a Case of Advanced Pulpal Necrosis and Apical Periodontitis. J. Endod..

[30] Zhao H., Feng J., Seidel K., Shi S., Klein O., Sharpe P., Chai Y. (2014). Secretion of shh by a neurovascular bundle niche supports mesenchymal stem cell homeostasis in the adult mouse incisor. Cell Stem Cell.

[31] Men Y., Wang Y., Yi Y., Jing D., Luo W., Shen B., Stenberg W., Chai Y., Ge W.P., Feng J.Q. (2020). Gli1+ Periodontium Stem Cells Are Regulated by Osteocytes and Occlusal Force. Dev. Cell.

[32] Diogenes A., Henry M.A., Teixeira F.B., Hargreaves K.M. (2013). An update on clinical regenerative endodontics. Endod. Top..

[33] Diogenes A., Hargreaves K.M. (2017). Microbial Modulation of Stem Cells and Future Directions in Regenerative Endodontics. J. Endod..

[34] Scadden D.T. (2006). The stem-cell niche as an entity of action. Nature.

[35] Jones D.L., Wagers A.J. (2008). No place like home: Anatomy and function of the stem cell niche. Nat. Rev. Mol. Cell Biol..

[36] Yuan Y., Loh Y.-H.E., Han X., Feng J., Ho T.-V., He J., Jing J., Groff K., Wu A., Chai Y. (2020). Spatiotemporal cellular movement and fate decisions during first pharyngeal arch morphogenesis. Sci. Adv..

[37] Han X., Feng J., Guo T., Loh Y.-H.E., Yuan Y., Ho T.-V., Cho C.K., Li J., Jing J., Janeckova E. (2021). Runx2-Twist1 interaction coordinates cranial neural crest guidance of soft palate myogenesis. eLife.

[38] Joo K.H., Song J.S., Kim S., Lee H.S., Jeon M., Kim S.O., Lee J.H. (2018). Cytokine Expression of Stem Cells Originating from the Apical Complex and Coronal Pulp of Immature Teeth. J. Endod..

[39] Somoza R.A., Acevedo C.A., Albornoz F., Luz-Crawford P., Carrión F., Young M.E., Weinstein-Oppenheimer C. (2017). TGF*β*3 secretion by three-dimensional cultures of human dental apical papilla mesenchymal stem cells. J. Tissue Eng. Regen. Med..

[40] Xu X., Zheng L., Yuan Q., Zhen G., Crane J.L., Zhou X., Cao X. (2018). Transforming growth factor-β in stem cells and tissue homeostasis. Bone Res..

[41] Srisuwan T., Wattanapakkavong K. (2022). Direct effect of transforming growth factor-beta 1 (TGF-β1) on human apical papilla cell proliferation and mineralisation. Aust. Endod. J..

[42] Akhila A., Prabath Singh V., Varma K., Vasudevan S., Sukhithasri V., Sasikumar S. (2021). An in-vitro comparative evaluation of quantitative release of transforming growth factor β-1 from dentin upon the action of endodontic irrigants, medicaments, ultrasonic activation, and low-level laser irradiation. Amrita J. Med..

[43] Chae Y., Yang M., Kim J. (2018). Release of TGF-β1 into root canals with various final irrigants in regenerative endodontics: An in vitro analysis. Int. Endod. J..

[44] He W., Zhang J., Niu Z., Yu Q., Wang Z., Zhang R., Su L., Fu L., Smith A.J., Cooper P.R. (2014). Regulatory Interplay between NFIC and TGF-β1 in Apical Papilla-derived Stem Cells. J. Dent. Res..

[45] Li J., Ge L., Zhao Y., Zhai Y., Rao N., Yuan X., Yang J., Li J., Yu S. (2022). TGF-β2 and TGF-β1 differentially regulate the odontogenic and osteogenic differentiation of mesenchymal stem cells. Arch. Oral Biol..

[46] Yu S., Li J., Zhao Y., Li X., Ge L. (2020). Comparative Secretome Analysis of Mesenchymal Stem Cells from Dental Apical Papilla and Bone Marrow during Early Odonto/Osteogenic Differentiation: Potential Role of Transforming Growth Factor-β2. Front. Physiol..

[47] Zhang W., Zhang X., Ling J., Liu W., Zhang X., Ma J., Zheng J. (2014). Proliferation and odontogenic differentiation of BMP2 gene-transfected stem cells from human tooth apical papilla: An in vitro study. Int. J. Mol. Med..

[48] Wang W., Dang M., Zhang Z., Hu J., Eyster T.W., Ni L., Ma P.X. (2016). Dentin regeneration by stem cells of apical papilla on injectable nanofibrous microspheres and stimulated by controlled BMP-2 release. Acta. Biomater..

[49] Diao S., Lin X., Wang L., Dong R., Du J., Yang D., Fan Z. (2017). Analysis of gene expression profiles between apical papilla tissues, stem cells from apical papilla and cell sheet to identify the key modulators in MSCs niche. Cell Prolif..

[50] Wang J., Liu B., Gu S., Liang J. (2012). Effects of Wnt/β-catenin signalling on proliferation and differentiation of apical papilla stem cells. Cell Prolif..

[51] Jin L., Cao Y., Yu G., Wang J., Lin X., Ge L., Du J., Wang L., Diao S., Lian X. (2017). SFRP2 enhances the osteogenic differentiation of apical papilla stem cells by antagonizing the canonical WNT pathway. Cell. Mol. Biol. Lett..

[52] Yang H., Li G., Han N., Zhang X., Cao Y., Cao Y., Fan Z. (2020). Secreted frizzled-related protein 2 promotes the osteo/odontogenic differentiation and paracrine potentials of stem cells from apical papilla under inflammation and hypoxia conditions. Cell Prolif..

[53] Fu Y., Ma D., Fan F., Sun T., Han R., Yang Y., Zhang J. (2022). Noncanonical Wnt5a Signaling Suppresses Hippo/TAZ-Mediated Osteogenesis Partly through the Canonical Wnt Pathway in SCAPs. Drug Des. Dev. Ther..

[54] Mossahebi-Mohammadi M., Quan M., Zhang J.-S., Li X. (2020). FGF signaling pathway: A key regulator of stem cell pluripotency. Front. Cell Dev. Biol..

[55] Chang M.-C., Chen C.-Y., Chang Y.-C., Zhong B.-H., Wang Y.-L., Yeung S.-Y., Chang H.-H., Jeng J.-H. (2020). Effect of bFGF on the growth and matrix turnover of stem cells from human apical papilla: Role of MEK/ERK signaling. J. Formos. Med. Assoc..

[56] Wu J., Huang G.T., He W., Wang P., Tong Z., Jia Q., Dong L., Niu Z., Ni L. (2012). Basic fibroblast growth factor enhances stemness of human stem cells from the apical papilla. J. Endod..

[57] Fayazi S., Takimoto K., Diogenes A. (2017). Comparative Evaluation of Chemotactic Factor Effect on Migration and Differentiation of Stem Cells of the Apical Papilla. J. Endod..

[58] Chang M.-C., Chen N.-Y., Chen J.-H., Huang W.-L., Chen C.-Y., Huang C.-C., Pan Y.-H., Chang H.-H., Jeng J.-H. (2021). bFGF stimulated plasminogen activation factors, but inhibited alkaline phosphatase and SPARC in stem cells from apical Papilla: Involvement of MEK/ERK, TAK1 and p38 signaling. J. Adv. Res..

[59] Wang S., Mu J., Fan Z., Yu Y., Yan M., Lei G., Tang C., Wang Z., Zheng Y., Yu J. (2012). Insulin-like growth factor 1 can promote the osteogenic differentiation and osteogenesis of stem cells from apical papilla. Stem Cell Res..

[60] Cui Y., Bai M., Guo D., Yang Y., Chen H., Sun J., Xie J., Zhou X. (2021). Insulin-like growth factor 1 promotes neural differentiation of human stem cells from the apical papilla. Arch. Oral Biol..

[61] Diao S., Yang H., Cao Y., Yang D., Fan Z. (2020). IGF2 enhanced the osteo-/dentinogenic and neurogenic differentiation potentials of stem cells from apical papilla. J. Oral Rehabil..

[62] Zhang W., Zhang X., Ling J., Wei X., Jian Y. (2016). Osteo-/odontogenic differentiation of BMP2 and VEGF gene-co-transfected human stem cells from apical papilla. Mol. Med. Rep..

[63] Shen Z., Tsao H., LaRue S., Liu R., Kirkpatrick T.C., Souza L.C., Letra A., Silva R.M. (2021). Vascular Endothelial Growth Factor and/or Nerve Growth Factor Treatment Induces Expression of Dentinogenic, Neuronal, and Healing Markers in Stem Cells of the Apical Papilla. J. Endod..

[64] Yan M., Wu J., Yu Y., Wang Y., Xie L., Zhang G., Yu J., Zhang C. (2014). Mineral trioxide aggregate promotes the odonto/osteogenic differentiation and dentinogenesis of stem cells from apical papilla via nuclear factor kappa B signaling pathway. J. Endod..

[65] Forrester S.J., Booz G.W., Sigmund C.D., Coffman T.M., Kawai T., Rizzo V., Scalia R., Eguchi S. (2018). Angiotensin II Signal Transduction: An Update on Mechanisms of Physiology and Pathophysiology. Physiol. Rev..

[66] Pizzatto L.N., Meneses C.C., Diniz E.A., Dionísio T.J., Santos C.F., Sipert C.R. (2020). Angiotensin II regulates proliferation and function of stem cells of apical papilla. J. Endod..

[67] Xiao M., Qiu J., Kuang R., Zhang B., Wang W., Yu Q. (2019). Synergistic effects of stromal cell-derived factor-1α and bone morphogenetic protein-2 treatment on odontogenic differentiation of human stem cells from apical papilla cultured in the VitroGel 3D system. Cell Tissue Res..

[68] Fehrmann C., Dörfer C.E., El-Sayed K.M.F. (2020). Toll-like receptor expression profile of human stem/progenitor cells form the apical papilla. J. Endod..

[69] Petridis X., van der Sluis L.W., Dijkstra R.J., Brinker M.G., van der Mei H.C., Harmsen M.C. (2018). Secreted products of oral bacteria and biofilms impede mineralization of apical papilla stem cells in TLR-, species-, and culture-dependent fashion. Sci. Rep..

[70] Lei S., Liu X.-M., Liu Y., Bi J., Zhu S., Chen X. (2020). Lipopolysaccharide Downregulates the Osteo-/Odontogenic Differentiation of Stem Cells from Apical Papilla by Inducing Autophagy. J. Endod..

[71] Lertchirakarn V., Aguilar P. (2017). Effects of lipopolysaccharide on the proliferation and osteogenic differentiation of stem cells from the apical papilla. J. Endod..

[72] Vishwanat L., Duong R., Takimoto K., Phillips L., Espitia C.O., Diogenes A., Ruparel S.B., Kolodrubetz D., Ruparel N.B. (2017). Effect of Bacterial Biofilm on the Osteogenic Differentiation of Stem Cells of Apical Papilla. J. Endod..

[73] Rakhimova O., Schmidt A., Landström M., Johansson A., Kelk P., Romani Vestman N. (2020). Cytokine Secretion, Viability, and Real-Time Proliferation of Apical-Papilla Stem Cells Upon Exposure to Oral Bacteria. Front. Cell. Infect. Microbiol..

[74] Wang J., Dai J., Liu B., Gu S., Cheng L., Liang J. (2013). Porphyromonas gingivalis lipopolysaccharide activates canonical Wnt/β-catenin and p38 MAPK signalling in stem cells from the apical papilla. Inflammation.

[75] Miron P.-O., Ben Lagha A., Azelmat J., Diogenes A., Nascimento Santos J., Grenier D. (2021). Production of TNF-*α* by macrophages stimulated with endodontic pathogens and its effect on the biological properties of stem cells of the apical papilla. Clin. Oral Investig..

[76] Zymovets V., Razghonova Y., Rakhimova O., Aripaka K., Manoharan L., Kelk P., Landström M., Romani Vestman N. (2022). Combined Transcriptomic and Protein Array Cytokine Profiling of Human Stem Cells from Dental Apical Papilla Modulated by Oral Bacteria. Int. J. Mol. Sci..

[77] Martin D.E., De Almeida J.F.A., Henry M.A., Khaing Z.Z., Schmidt C.E., Teixeira F.B., Diogenes A. (2014). Concentration-dependent effect of sodium hypochlorite on stem cells of apical papilla survival and differentiation. J. Endod..

[78] Trevino E.G., Patwardhan A.N., Henry M.A., Perry G., Dybdal-Hargreaves N., Hargreaves K.M., Diogenes A. (2011). Effect of irrigants on the survival of human stem cells of the apical papilla in a platelet-rich plasma scaffold in human root tips. J. Endod..

[79] Widbiller M., Althumairy R.I., Diogenes A. (2019). Direct and indirect effect of chlorhexidine on survival of stem cells from the apical papilla and its neutralization. J. Endod..

[80] Ruparel N.B., Teixeira F.B., Ferraz C.C., Diogenes A. (2012). Direct effect of intracanal medicaments on survival of stem cells of the apical papilla. J. Endod..

[81] Althumairy R.I., Teixeira F.B., Diogenes A. (2014). Effect of dentin conditioning with intracanal medicaments on survival of stem cells of apical papilla. J. Endod..

[82] Raddall G., Mello I., Leung B.M. (2022). Effects of Intracanal Antimicrobials on Viability and Differentiation of Stem Cells from the Apical Papilla: An in Vitro Study. J. Endod..

[83] Miller A.A., Takimoto K., Wealleans J., Diogenes A. (2018). Effect of 3 bioceramic materials on stem cells of the apical papilla proliferation and differentiation using a dentin disk model. J. Endod..

[84] Wongwatanasanti N., Jantarat J., Sritanaudomchai H., Hargreaves K.M. (2018). Effect of bioceramic materials on proliferation and odontoblast differentiation of human stem cells from the apical papilla. J. Endod..

[85] Becerra P., Ricucci D., Loghin S., Gibbs J.L., Lin L.M. (2014). Histologic study of a human immature permanent premolar with chronic apical abscess after revascularization/revitalization. J. Endod..

[86] Zhou R., Wang Y., Chen Y., Chen S., Lyu H., Cai Z., Huang X. (2017). Radiographic, Histologic, and Biomechanical Evaluation of Combined Application of Platelet-rich Fibrin with Blood Clot in Regenerative Endodontics. J. Endod..

[87] Lovelace T.W., Henry M.A., Hargreaves K.M., Diogenes A. (2011). Evaluation of the delivery of mesenchymal stem cells into the root canal space of necrotic immature teeth after clinical regenerative endodontic procedure. J. Endod..

